# Supplementary material for: Proteomic Analysis of Human Milk Reveals Nutritional and Immune Benefits in the Colostrum from Mothers with COVID-19
Source: Nutrients. 2022 Jun 17;14(12):2513. doi: 10.3390/nu14122513 (PMC9227629; doi:10.3390/nu14122513)
Supplement: Supplementary file 1 [file nutrients-14-02513-s001.zip › Figure S1-6.pdf]

Supplementary Materials for manuscript entitled

Proteomic analysis of human milk reveals nutritional and immune benefits in the colostrum from mothers with COVID-19

Juanjuan Guo[a,d,#], Minjie Tan[b,#], Jing Zhu[c,✉], Ye Tian[b], Huanyu Liu[a,d], Fan Luo[e], Jianbin Wang[f], Yanyi Huang[b,g], Yuanzhen Zhang[a,d,h], Yuexin Yang[i], Guanbo Wang[b,g,✉]

[a] Department of Gynaecology and Obstetrics, Zhongnan Hospital of Wuhan University, Wuhan, Hubei Province 430071, China

[b] Institute for Cell Analysis, Shenzhen Bay Laboratory, Shenzhen, Guangdong Province 518132, China

[c] Institute of Biotechnology and Health, Beijing Academy of Science and Technology, 100089, Beijing, China

[d] Hubei Clinical Research Center for Prenatal Diagnosis and Birth Health, Wuhan 430071, China

[e] State Key Laboratory of Virology/Institute of Medical Virology, School of Basic Medical Sciences, Wuhan University, Wuhan 430071, China

[f] School of Life Sciences and Tsinghua-Peking Center for Life Sciences, Tsinghua University, Beijing 100084, China

[g] Biomedical Pioneering Innovation Center, Peking University, Beijing 100871, China

[h] Hubei Provincial Key Laboratory of Developmentally Originated Disease, Wuhan, Hubei Province 430071, China

[i] National Institute for Nutrition and Health, Chinese Center for Disease Control and Prevention, Beijing, 100050, China

# Equal contribution to this work

Table of Contents

Supplementary Figures……….. S2-S7

Supplementary Table Captions ………S8

✉ Jing Zhu, Email: jingzhu.nutri@outlook.com

✉ Guanbo Wang, Email: guanbo.wang@pku.edu.cn

SUPPLEMENTARY FIGURES

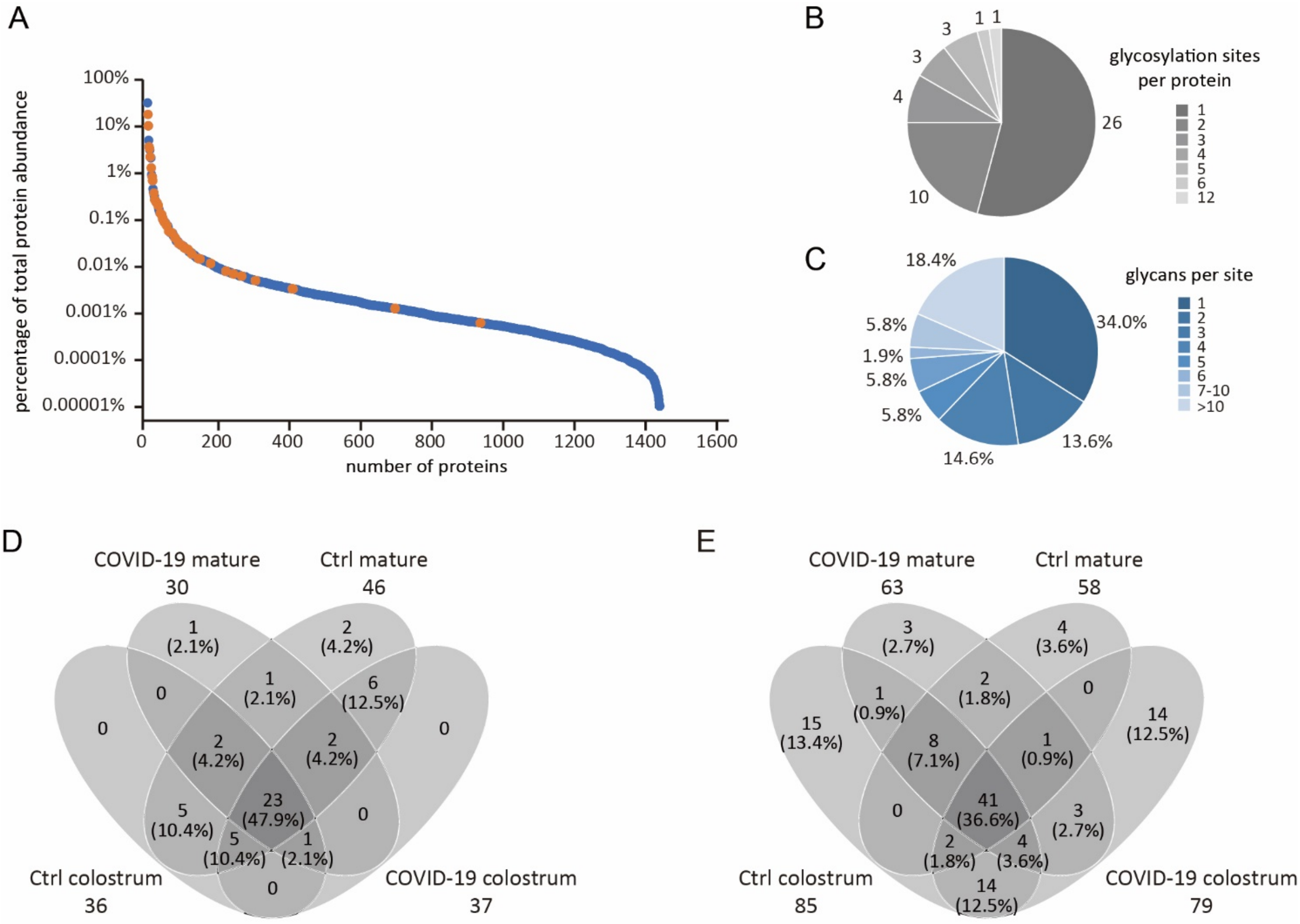

Figure S1. Overview of *N*-glycoproteomes in different sample groups. (**A**) Dynamic range of identified proteins. Orange dots indicate identified glycoproteins and blue dots represent proteins without identified glycosylation sites. (**B**) Distributions of the numbers of glycosylation sites detected at each identified glycoprotein. (**C**) Distributions of the glycosylation sites detected with different numbers of glycans. (**D** and **E**) Venn diagrams illustrating (**D**) the numbers of glycoproteins and (**E**) the numbers of glycopeptides identified in each sample group.

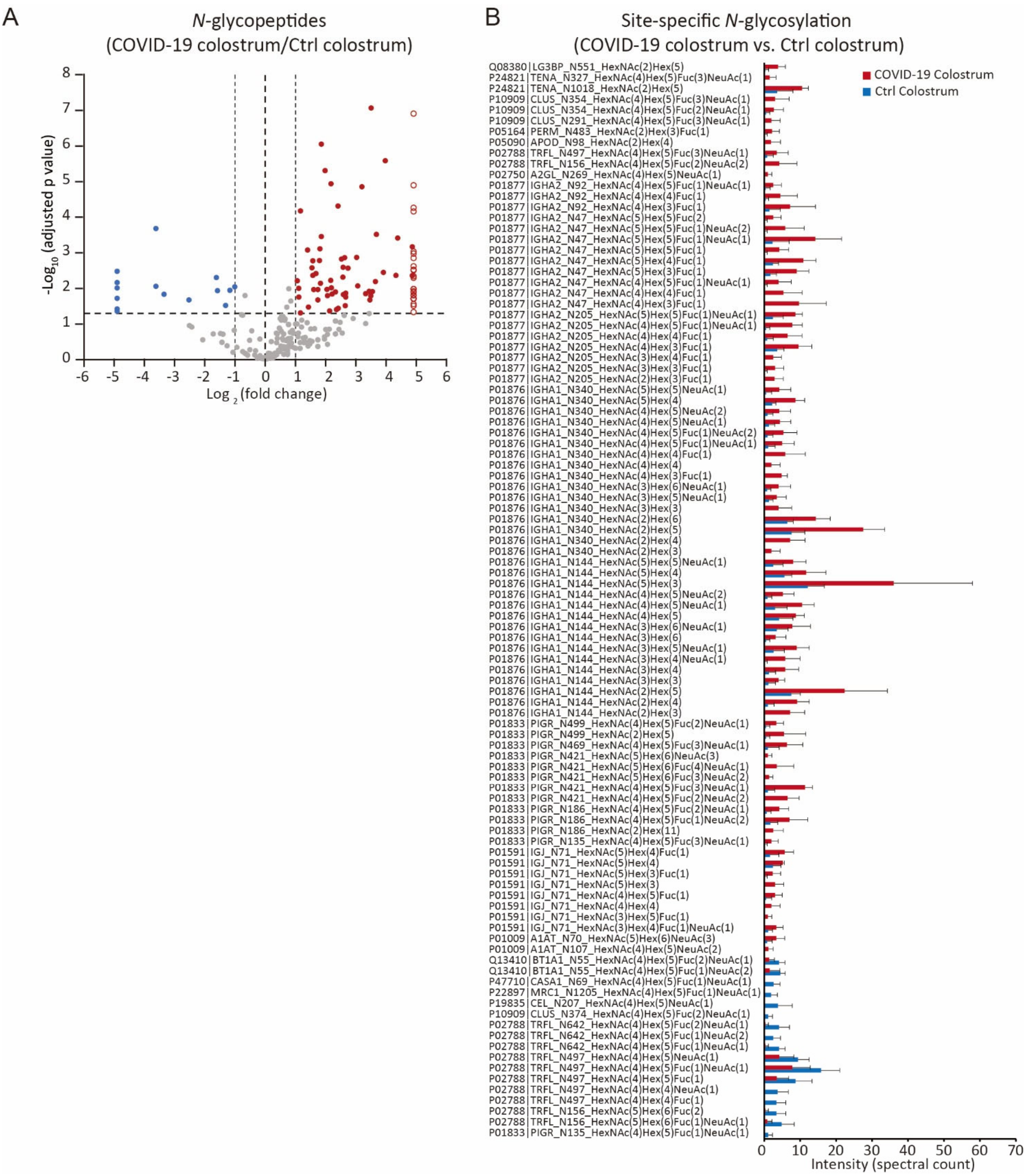

Figure S2. Comparison of *N*-glycoproteome profiles in the COVID-19 colostrum and Ctrl colostrum groups. (**A**) A volcano plot showing changes in glycopeptides. The open circles represent glycopeptides that were exclusively detectable in the COVID-19 colostrum group; their abundances in the COVID-19 mature group were manually set as a fixed minimum quantitative value, thereby giving rise to an identical fold-change value in the plot. (**B**) A bar graph showing changes in site-specific *N*-glycosylation. Bars indicate means and whiskers indicate SDs.

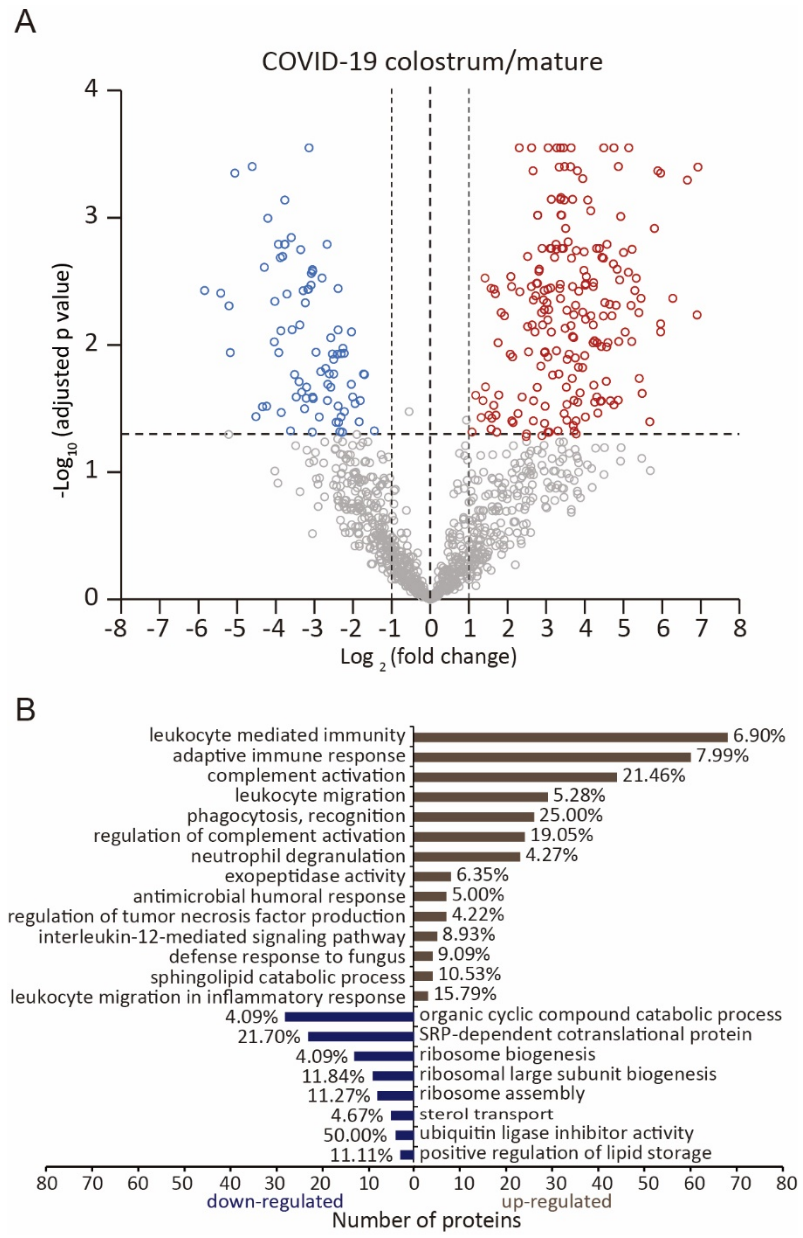

Figure S3. Comparison of COVID-19 colostrum and COVID-19 mature sample group proteomes. (**A**) A volcano plot of proteomes in COVID-19 colostrum and COVID-19 mature milk. (**B**) Enriched biological processes of differentially expressed proteins (p of each Gene Ontology [GO] term < 0.0002). The graph shows both numbers and percentages of differently expressed proteins in each term group.

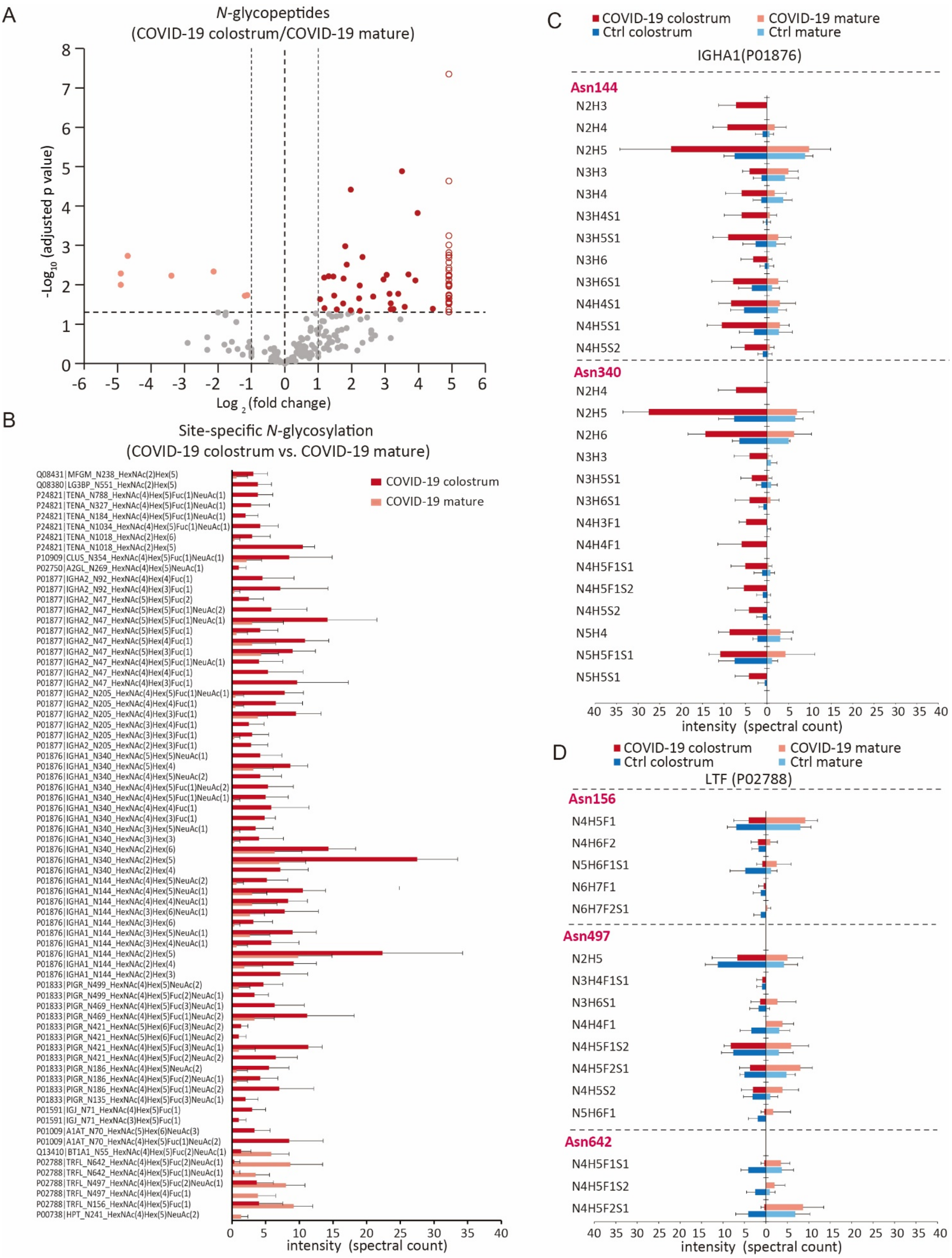

Figure S4. Comparisons of the *N*-glycoproteome profiles between the four sample groups. (**A**) A volcano plot showing changes in glycopeptides during milk maturation. The open circles represent glycopeptides that were

exclusively detectable in the COVID-19 colostrum group; their abundances in the COVID-19 mature group were manually set as a fixed minimum quantitative value, thereby giving rise to an identical fold-change value in the plot. (**B**) A bar graph showing changes in site-specific *N*-glycosylation during milk maturation. (**C**) Comparisons of site-specific immunoglobulin heavy constant alpha 1 (IGHA1) *N*-glycopeptides in the four sample groups. (**D**) Comparisons of site-specific lactoferrin (LTF) *N*-glycopeptides in the four sample groups. Bars indicate means and whiskers indicate SDs.

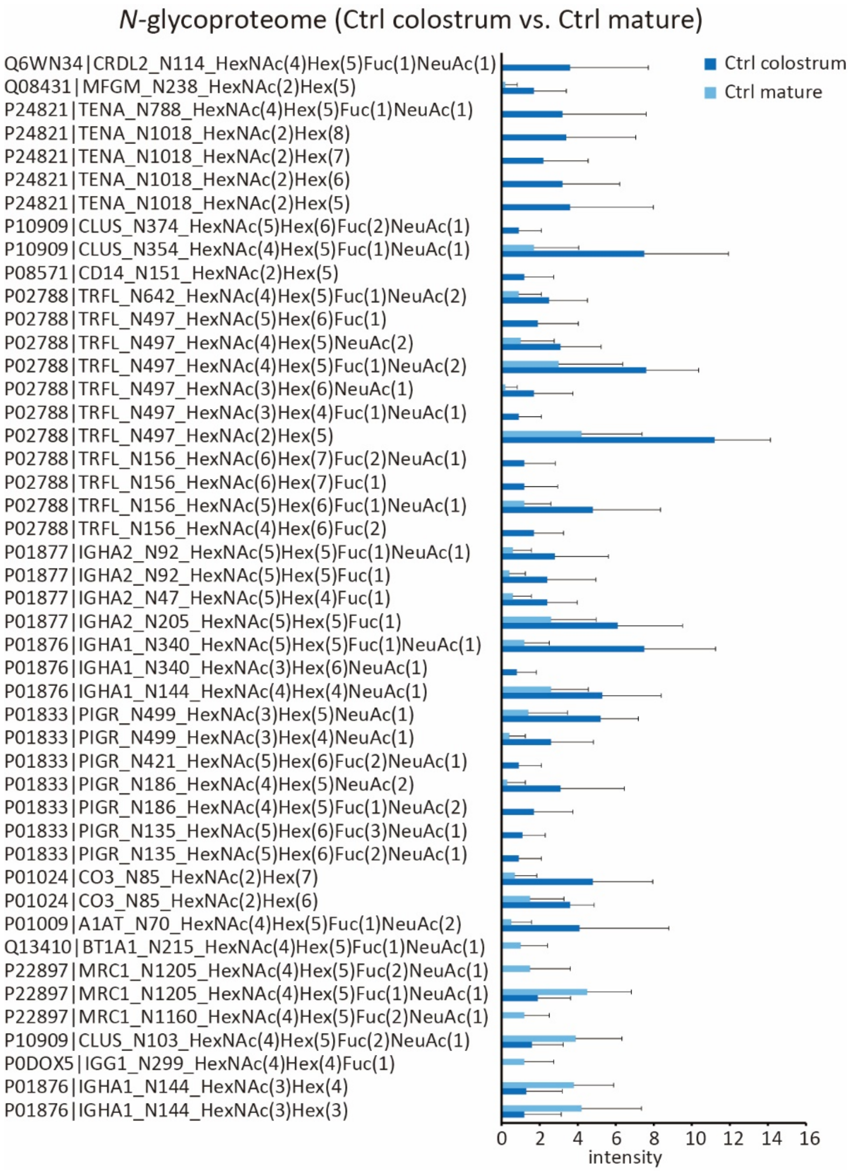

Figure S5. Changes in site-specific *N*-glycosylation during maturation of breastmilk from healthy women (Ctrl groups). Bars indicate means and whiskers indicate SDs.

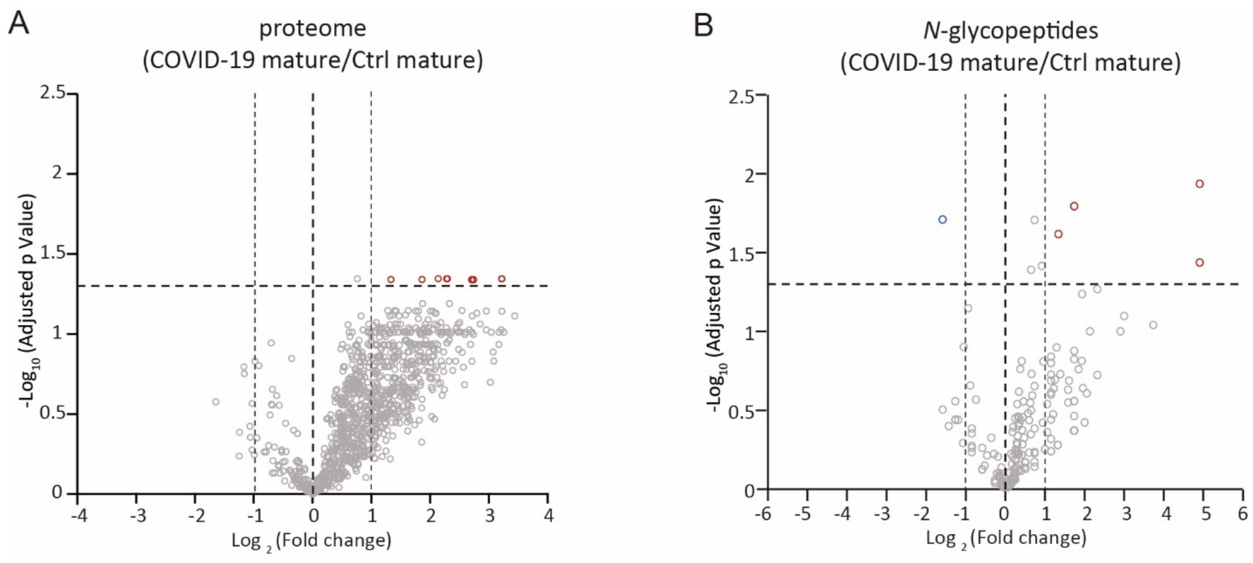

Figure S6. Comparisons of the proteome (**A**) and glycoproteome (**B**) profiles between mature milk samples from COVID-19 patients (COVID-19 mature) and healthy women (Ctrl mature). The red dots in the volcano plots indicate up-regulated (**A**) proteins and (**B**) *N*-glycopeptides in COVID-19 milk and the blue dots indicate the down-regulated ones.

SUPPLEMENTARY TABLE CAPTIONS

Table S1. List of proteins identified in all sample groups
Table S2. Enriched gene ontology (GO) terms associated with the differentially expressed proteins in COVID-19 colostrum and Ctrl colostrum sample groups
Table S3. Enriched GO terms associated with the differentially expressed proteins in COVID-19 colostrum and COVID-19 mature sample groups
Table S4. Peptide to spectrum matches (PSMs) of identified glycopeptides
Table S5. List of unique *N*-glycopeptides
